# Lipids of Prokaryotic Origin at the Base of Marine Food Webs

**DOI:** 10.3390/md10122698

**Published:** 2012-11-29

**Authors:** Carla C. C. R. de Carvalho, Maria José Caramujo

**Affiliations:** 1 IBB-Institute for Biotechnology and Bioengineering, Centre for Biological and Chemical Engineering, Department of Bioengineering, Instituto Superior Técnico, Technical University of Lisbon, Av. Rovisco Pais, Lisbon 1049-001, Portugal; 2 Centre for Environmental Biology, Faculty of Sciences, University of Lisbon, Campo Grande C2, Lisbon 1749-016, Portugal; Email: mj.caramujo@fc.ul.pt

**Keywords:** phospholipid, fatty acids, polyunsaturated fatty acids, extremophile, bacteria, archaea, trophic web

## Abstract

In particular niches of the marine environment, such as abyssal trenches, icy waters and hot vents, the base of the food web is composed of bacteria and archaea that have developed strategies to survive and thrive under the most extreme conditions. Some of these organisms are considered “extremophiles” and modulate the fatty acid composition of their phospholipids to maintain the adequate fluidity of the cellular membrane under cold/hot temperatures, elevated pressure, high/low salinity and pH. Bacterial cells are even able to produce polyunsaturated fatty acids, contrarily to what was considered until the 1990s, helping the regulation of the membrane fluidity triggered by temperature and pressure and providing protection from oxidative stress. In marine ecosystems, bacteria may either act as a sink of carbon, contribute to nutrient recycling to photo-autotrophs or bacterial organic matter may be transferred to other trophic links in aquatic food webs. The present work aims to provide a comprehensive review on lipid production in bacteria and archaea and to discuss how their lipids, of both heterotrophic and chemoautotrophic origin, contribute to marine food webs.

## 1. Introduction

The conditions of the marine environment led to the development of specialized lipid molecules responsible for the formation of membranes and storage of energy, and in higher organisms, for tissue formation, reproduction and growth. Fatty acids (FA) are important lipid compounds that are used as building blocks for the majority of lipid classes and as precursors for the biosynthesis of bioactive molecules. The fatty acid composition of phospholipids and their interaction with sterols and proteins determine the physical properties of cellular membranes, whilst neutral lipids, such as triacylglycerols (TAGs) and wax esters (WE), are reserves of fatty acids for energetic purposes and for phospholipid synthesis.

Marine bacteria use adaptive changes in lipid composition as a response to environmental variations in pressure, temperature and salinity [1]. Archaea, which constitute a significant fraction of the “picoplankton” in the dark ocean water below 150 m, and equal bacteria in numbers at depths greater than 1000 m [2], are adapted to life in extreme environments, such as hot vents, and contain a much more stable membrane than bacteria. Bacterial membranes contain phospholipids in which the fatty acid moieties are linked by ester bonds to glycerol and form a phospholipid bilayer. However, archaeal membranes have bipolar lipids containing two polar heads linked by isoprenoid chains and ether linkages to glycerol. This allows for the formation of a monolayer membrane, which is likely to be responsible for the ability of these cells to thrive in extreme environments. Other features distinguishing archaeal and bacterial lipids include: the glycerophosphate backbone of archaeal phospholipids is *sn*-glycerol-1-phosphate, while bacterial have a *sn*-glycerol-3-phosphate backbone; the majority of the isoprenoid hydrocarbon chains of polar lipids in archaea are methyl-branched, whilst their bacteria counterparts are mostly straight-chain fatty acids; and several archaeal species present bipolar lipids with a tetraether core [3].

The development of mass spectrometry equipment and techniques has enabled the determination of structure and function of lipids in living systems and the emergence of lipidomics as an important part of metabolomics. There are two approaches in mass spectrometry-based lipidomics: one is based on a separation of lipids into different classes prior to analysis, whilst the other uses a shotgun approach in which all lipid species are analyzed simultaneously without a prior separation [4,5]. Novel lipid species can also be discovered by operating mass spectrometers in full-scan mode to search for new mass-to-charge ratio peaks [4]. In the field of marine lipidology, these different approaches can be used to search for new fatty acid structures and new sources of polyunsaturated fatty acids (PUFAs), in studying the role of the several fatty acids in cell membranes and their biosynthetic pathways and in finding the best fatty acid biomarkers or fatty acid ratios to assess trophic transfer in ecosystems [6]. 

The recognition that PUFAs, especially docosahexaenoic (22:6ω3 or DHA) and eicosapentaenoic (20:5ω3 or EPA) acids, are fundamental to promote human health, by helping brain function and preventing cardiovascular diseases, increased the interest in these fatty acids [7,8,9]. The current global market for ω3 fatty acids is estimated to be 15,000–20,000 tons, derived from an approximate world production of fish oil of 300,000 tons per year [10]. Recent studies have shown that marine phospholipids have a better bioavailability, resistance to oxidation and a higher content of EPA and DHA than oily triglycerides from the same source [11,12]. Until the 1990s, it was considered that bacteria had no PUFA, with the exception of selected cyanobacteria. As noted by Okuyama *et al.* [13], such assumption may have resulted from the fact that the bacterial species whose physiology, biochemistry and molecular biology had been well studied until that time were mesophilic species, such as *Escherichia coli*, which have no PUFA. Additionally, the culture conditions may determine the bacterial EPA content that is dependent on pH, temperature and other growth conditions [14]. It is now accepted that some species have the capacity to produce EPA, DHA or arachidonic acid (20:4ω6, ARA) [15]. As pointed out in a review by Valentine and Valentine, PUFAs have significant structural roles in bacterial membranes, including: regulatory function triggered by temperature and pressure; EPA-enriched membranes support a respiratory lifestyle dependent on proton bioenergetics; and contribution to increased fluidity of the cellular membrane under marine conditions [16]. DHA and EPA should also protect the marine organisms from biotic and abiotic oxidative stresses caused by reactive oxygen species (ROS), which are prevalent in marine environments [17]. Although, polyunsaturated fatty acids, such as EPA and DHA, are very susceptible to oxygen and ROS, several studies indicate that these molecules are rather stable against oxidative stresses when they are *in vivo* [17,18,19].

The concept of FA being transferred conservatively through aquatic food webs and of their use as biomarkers was first suggested in 1935 by Lovern [20] and applied to trace the diet in marine environments in the 1960s by Ackman and Eaton. As a result, FA biomarker analyses have become an important tool for resolving trophic interactions in marine ecosystems [21]. The use of bacterial fatty acids or bacterial fatty acid ratios may thus be used to disclose bacterial connections to the marine food web and its importance to supply materials and energy to the higher trophic levels.

## 2. Lipid Production in Marine Micro-Organisms

Bacteria must be able to maintain the biological functions and integrity of the cellular membrane under stressful conditions, as this structure is responsible for controlling the entrance of solutes in the cell, for the maintenance of energy status, for signal transduction and for keeping turgor pressure. Lipids play an important role in the maintenance of cell viability under stressful conditions, as membrane fluidity is maintained by alterations in the fatty acid composition of the membrane phospholipids through a mechanism called “homeoviscous adaptation” [22]. However, several other lipid molecules, such as extracellular glycolipids, energy storage molecules, such as triacylglicerides, and defense lipids with antibacterial properties to fight competitors are also of paramount importance in marine environments.

### 2.1. Lipids as Protecting Agents in Marine Environments

Some of the marine environments, including hot vents, polar icy waters, acidic and alkaline waters, salt brines and pressurized abyssal trenches, present conditions so hostile to humans that they were initially considered too extreme to support microbial life. However, as sampling and laboratorial culture conditions techniques evolved, it was found that microbial “extremophiles” could survive and thrive in such environments [23]. They were named according to their optimal growth conditions as thermophiles (T_opt_ > 60 °C), hyperthermophiles (T_opt_ > 80 °C), psychrophiles (T_opt_ < 15 °C), acidophiles (pH_opt_ < 3), alkaliphiles (pH_opt_ > 8.5), halophiles (NaCl > 3%) and barophiles, or piezophiles. A few bacterial strains can endure both elevated temperature and extreme pH, being considered poly-extremophiles [24].

Extremophiles can be (i) obligate extremophiles, which only grow under one or more extreme conditions, and (ii) facultative extremophiles, which grow optimally at a non-extreme condition but can tolerate and thrive under conditions that are lethal or toxic to the majority of living organisms. Extremophiles present alterations in fatty acid composition of the cellular membranes and produce specialized lipids, allowing them to survive under conditions that kill most of the other micro-organisms. 

Most of the marine environment is characterized by a temperature below 4 °C and pressure above 100 × 10^5^ Pa, favoring psychrophilic and barophilic bacteria [25]. During a one-year study with monthly sampling throughout the water column (from surface to 4750 m deep) in Hawaii, it was found that pelagic crenarchaeota, a group of archaea, was equivalent in cell numbers to bacteria at depths greater than 1000 m [2]. The authors estimated that the global oceans harbor approximately 1.3 × 10^28^ archaeal cells and 3.1 × 10^28^ bacterial cells. Biogeochemical and stable carbon isotopic analyses of a sedimentary record of archaeal lipids indicate that an anoxic event in Earth history led certain hyperthermophilic Archaea to adapt to low-temperature environments and to their massive expansion [26]. In subsurface sediments, buried deeper than 1 m in a wide range of oceanographic settings, it was found that at least 87% of intact polar membrane lipids could be attributable to archaeal membranes [27].

**Figure 1:**
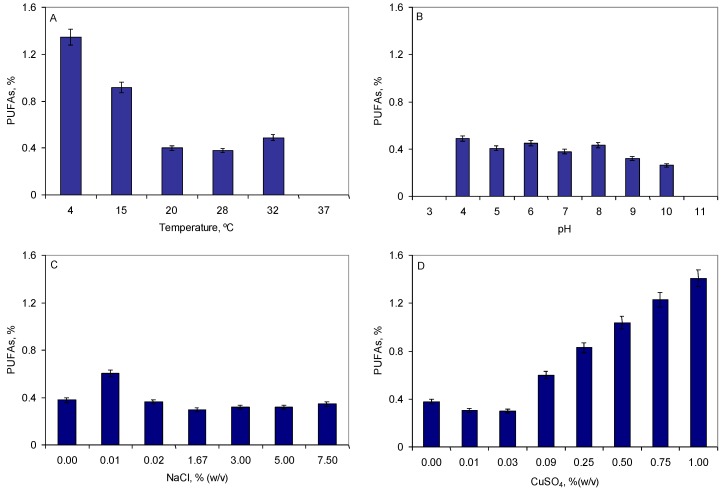
Production of polyunsaturated fatty acids in *R. erythropolis* during adaptation to temperature (**A**), pH (**B**), high concentration of salt (**C**) or copper sulphate (**D**).

The fatty acid composition of the membrane phospholipids regulates membrane fluidity. At extremely low temperatures, an increase in the content of unsaturated and polyunsaturated fatty acids and a decrease in the average chain length of fatty acids in cellular membranes is observed [28,29]. Several psychrophilic bacterial strains isolated from sea ice produce a novel enzyme family required for the biosynthesis of PUFAs at low temperatures called polyketide synthase (PKS) [30,31]. The genes encoding these enzymes responsible for *de novo* long-chain PUFA biosynthesis are designated *pfaEABCD* and were thought to exist in the narrow subset of marine bacteria able to produce long-chain fatty acids [32]. However, the genetic potential to produce long-chain fatty acids via a FAS/PKS mechanism seems to be scattered throughout the bacterial domain [32]. During a stepwise adaptation of *Rhodococcus erythropolis* DCL14 cells from optimal growth conditions (28 °C, pH 7.0) to extreme conditions (that previously killed non-adapted cells) [13], it was found that the cells produced increased amounts of polyunsaturated fatty acids at lower temperatures and in the presence of copper sulphate (Figure 1). The cells produced 2.4 and 3.6 times more PUFAs at 15 and 4 °C, respectively, than at 28 °C (Figure 1A). A dose-dependent increase in the content of PUFAs was observed with copper sulphate for concentrations higher than 0.03% (w/v), reaching a 3.7-fold increase at 1% (Figure 1D). The pH and salt concentration did not significantly affected PUFA production in *R. erythropolis* (Figure 1B,C). A type Q gene cluster homologous to the *pfa* genes had been found in *R. erythropolis* PR4 [32].

Lipids are known to be heat-sensitive, and hyperthermophile bacteria produce special lipids. The bacterium *Thermotoga maritima*, which presents one of the highest growth temperatures at 90 °C, contains a novel glycerol ether lipid called 15,16-dimethyl-30-glyceryloxy-triacontanedioic acid that confers protection against hydrolysis at high temperatures [33]. The lipids of thermophilic archaea are characterized by unique structural features: they contain isoprenoid (phytanyl) chains with 15, 20, 25 or 40 carbons instead of straight chains observed in other organisms; two of these chains are linked *via* ether linkages to glycerol or a polyol; the glycerol found in archaea, 2,3-di-*O*-*sn*-glycerol, has the reverse stereochemistry when compared to that found in other organisms [34]. The ether lipids derived from diphytanyl-glycerol, or from its dimer di(biphytanyl)-diglycerol, are present on all archaeal membranes and confer to the cells a remarkable resistance against hydrolysis at high temperatures and acidic pH [35]. Furthermore, at high temperatures, an increased degree of cyclization of the aliphatic core of archaeal membranes is observed with a larger ratio of tetraether lipids *vs.* diether lipids, and the number of cyclopentane rings can vary up to four per aliphatic chain, thus maintaining membrane fluidity [36,37].

At high temperatures, thermophiles have problems keeping the intracellular concentration of Na^+^, since at a high temperature, the cell membrane becomes more permeable to the diffusion of protons and sodium ions [38]. Higher salinity concentrations cause, in general, an increase in the content of negatively charged phospholipids at the expense of neutral phospholipids. Gram-negative bacteria decrease the proportion of zwitterionic phosphatidylethanolamine in the membrane, while increasing the proportion of negatively charged phosphatidylglycerol and/or diphosphatidylglycerol [39,40]. In gram-positive bacteria, the anionic lipid fraction increases with salinity as a result of a higher content of diphosphatidylglycerol rather than phosphatidylglycerol [40].

When the transcriptional profiling of the halophile *Halobacterium* sp. NRC-1, which was among the first Archaea to be completely sequenced, was studied, it was found that growth at cold temperatures altered the expression of genes involved in lipid metabolism [41]. The gene coding for *sn*-1-glycerol phosphate dehydrogenase, responsible for the first step in the synthesis of polar lipids, was down regulated by 2.7-fold, while up-regulation was observed in the genes encoding for dehydrogenases for increased turnover of polar lipids, for a long-chain fatty acid-CoA ligase and for acetoacetyl-CoA thiolase, allowing the strain to alter the composition in lipids in the cold.

The fluidizing properties of EPA/DHA on cellular membranes seem a key point in barophilic bacteria, which have to carry out respiration at temperatures near 0 °C and under extremely high hydrostatic pressure. In *Acholeoplasma laidlawii*, when only 50% of the total lipids are in the fluid state, bacteria can still slowly grow and replicate, but growth ceases when around 90% of the membrane lipids pass from the liquid crystalline to the gel phase [42]. Although DHA and EPA phospholipids have an important role in disrupting or blocking the formation of islands of gel-phase lipids, there could be more fluidizing processes or lipids involved [15]. 

Two barophilic bacteria isolated from sediments from the Marianas Trench, DB21MT-2 and DB21MT-5, presented novel phospholipids in the classes of phosphatidylglycerol (PG) and phosphatidylethanolamine (PE) and its derivatives, phosphatidylmethylethanolamine (PME) and phosphatidyldimethylethanolamine (PDME) [43]. The phospholipids contained a high amount of 20:5ω3 (EPA; in DB21MT-2) and 22:6ω3 (DHA; in both strains) on the *sn*-1 and mostly on the *sn*-2 position of the phospholipids. Furthermore, the PUFAs were associated with almost every PG molecule, which was expected to cause greater disruption in acyl chain packing due to the larger head group of this phospholipid. The studies by Fang *et al.* [43] also suggested that psychrophilic and barophilic bacteria should be the major contributors of PUFAs to deep-sea sediments, since the vertical flux of PUFAs from surface water plankton decrease rapidly with depth.

### 2.2. Production of Specialized Lipids

The marine environment has favored the production of unique fatty acids and lipid molecules (Table 1). Bacterial fatty acids that can be used as biomarkers in marine environment are typically odd-numbered, branched *trans*-unsaturated and cyclopropyl fatty acids, e.g., 15:0, 17:0, 10-methyl-16:0, *iso*- and *anteiso*-branched saturated and monounsaturated [5]. Besides phospholipids, fatty acids are the building blocks of other lipid classes, including ceramides, wax esters, glycosphingolipids and *N*-acylated lipid molecules. Cyanobacteria are a source of acylated lipids and fatty acid amides [5]. The marine cyanobacterium *Oscillatoria* sp. produces a new diacylgalactolipid comprising 9,12-octadecadienoyl and 4-hexadecenoyl chains [44], whilst *Lyngbya majuscule* produces bioactive secondary malyngamides, such as Malyngamide G and 7-methoxydodec-4(*E*)-enoic acid [45,46].

Extremophiles are also good sources of unusual fatty acids. Psychrophilic *Bacillus* species produce relatively rare Δ^5^-isomers, although no obvious advantage for growth at low temperature is provided by these isomers when compared to membrane lipids with Δ^9^- or Δ^11^-isomers [47]. *Bacteroides fragilis *produces a branched-chain hydroxyl fatty acid in the amide for 3-hydroxy-15-methylhexadecanoic acid in lipopolysaccharides, which is rather specific in gram-negative bacteria [48]. The archaea *Thermoplasma acidophilum*, whose optimal growth conditions are 55–59 °C and pH 1–2, produces a peculiar membrane with 82% polar lipids having as the main polar lipid a bipolar tetraether lipid with a phosphoglycerol and a β-L-gulopyranose as head groups and up to four cyclopentane rings per aliphatic chain [37].

**Table 1:** Unusual lipids produced by micro-organisms.

**Bacteria**			
*Thermotoga maritima*	15,16-dimethyl-30-glyceryloxy-triacontanedioic acid	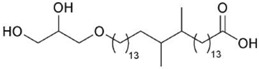	[33]
Bacteria from fish microbiome	sebastenoic acid	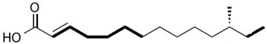	[49]
Marine bacteria such as *Shewanella putrefaciens*	furan-acids	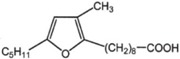	[50,51]
*Bacillus* sp.	ω-cycloheptane fatty acids	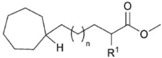	[52]
**Cyanobacteria**			
*Lyngbya majuscula *	malyngamide G	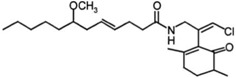	[45]
**Archaea**			
*Thermoplasma acidophilum*	main polar lipid	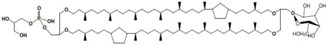	[37]
*Halobacterium salinarum*	2,3-diphytanyl-*sn*-glycerol-1-phospho- 3′-*sn*-glycerol-1′-methylphosphate	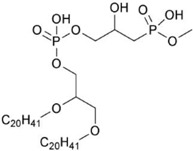	[53]

In a recently published paper, Sanchez *et al*. [49] examined for the first time a fish microbiome to isolate bacteria able to produce unique marine natural products. The fish intestines were a source of *Actinomycetales*, as well as unique strains of *Firmicutes* and *Proteobacteria*. The chemical extracts contained a new bioactive lipid called sebastenoic acid, which has anti-microbial activity against *Staphylococcus aureus*, *Bacillus subtilis*, *Enterococcus faecium* and *Vibrio mimicus*.

Furan fatty acids, shown to be scavengers of hydroxyl and peroxyl radicals and to provide potential protective properties in mammalian tissue and blood, have been found in, e.g., marine sponges, algae, plants and also in marine bacteria, such as *Shewanella putrefaciens* [50,51]. These acids are tri- or tetra-substituted furan derivatives characterized by either a propyl or pentyl side chain in one of the α-positions and a substituted straight long-chain saturated acid with a carboxylic group at its end on the other.

### 2.3. Non-Polar Phospholipids, Non-Phosphorous Polar Lipids and Neutral Lipids

The analyses of phospholipid-based fatty acids (PLFAs) were introduced as a means to assess live bacterial biomass, since they are rapidly degraded after cell death [21]. However a surface sediment from Carteau cove, France, contained, apart from phospholipids, non-phospholipid polar compounds with 12- to 28-carbon atoms, which cautions against the use of PLFAs to assess bacterial biomass without preliminary analysis and purification of phospholipids [54]. 

Some bacteria incorporate fatty acids containing furan in their phospholipids. Among the species able to produce furan acids are *Shewanella putrefaciens*, *Marinomonas comunis*, *Enterobacter agglomerans* and *Pseudomonas fluorescens*, which were isolated from the intestinal liquor of fishes [55]. It was proposed that in marine bacteria living in fish, furan acids are generated by incorporation of a methyl group into *cis*-vaccenic acid, followed by introduction of a second double bond, and the diunsaturated fatty acid formed is presumed to react with oxygen, followed by ring closure, to assume the final furan acid structure [51]. These acids present a radical-scavenging ability and should help in protecting the cells [50].

Marine oil-degrading or hydrocarbonoclastic bacteria usually produce significant amounts of neutral lipids, which can be used as storage compounds, probably as a result of sporadic availability of hydrocarbons as growth substrates [56,57]. Among these compounds are triacylglycerols, diacylglycerols, wax esters and polyhydroxyalkanoates. The marine hydrocarbonoclastic bacterium *Marinobacter* sp. PAD-2 produced extracellular wax ester-like compounds when grown on hexadecane or succinate as the sole carbon source [57]. When Alvarez *et al.* tested forty psychrophile or psychrotrophic crude oil-utilizing bacteria, they found that around 73% of the strains were able to accumulate specialized lipids, such as polyhydroalkanoic acids (PHAs), and two strains were able to produce wax esters as storage compounds [56]. PHA accumulation was predominantly observed between 4 and 20 °C.

**Figure 2:**
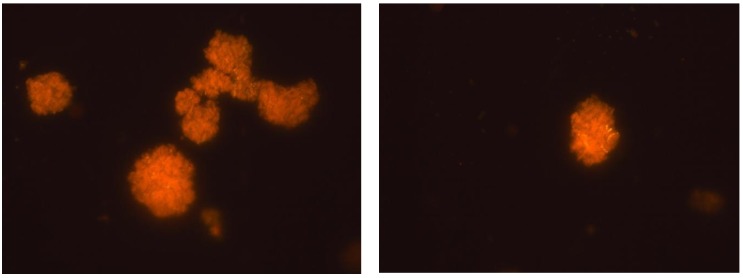
Nile Red staining of extracellular glycolipids produced by *R. erythropolis* during dehydration.

A *R. erythropolis* strain, able to degrade hydrocarbons and fuel oil under saline conditions [58], produces and excretes a trehalose based glycolipid to increase the bioavailability of hydrocarbons with reduced water solubility, and also when the cells are dehydrated (Figure 2). Extracellular polymeric substances provide protection for microbial cells, resulting in increased resilience under stressful periods [59]. Among the best examples of temporary stresses are marine intertidal conditions. In this case, micro-organisms are mainly in immobilized communities called biofilms, which confer protection against high temperature and exposure to ultraviolet radiation, temporary dehydration, limited access to nutrients and competition [60]. Curiously, intertidal bacteria have also been found to be a good source of PUFAs, with *Shewanella colwelliana*, *Vibrio splendidus* and *Photobacterium lipolyticum* being isolated from anoxic intertidal sediments [61].

## 3. Transfer of Bacterial Lipids to Metazoans in Marine Foodwebs

Bacteria may either act as a sink of carbon in aquatic ecosystems, contribute to nutrient recycling to autotrophs or bacterial organic matter may be transferred to other trophic links in aquatic foodwebs [62]. Either as the sole diet or associated with other dietary items, bacteria are ingested by small sized aquatic animals. Although concentrations of bacteria-derived FA, including odd-numbered saturated, branched-chain and monounsaturated (e.g., 18:1ω7), are retained by macrozooplankton [20], the ecological significance of the bacterial FA retention in crustacean consumers is still unclear. The low dietary value of bacteria relative to that of photosynthetic autotrophs (e.g., diatoms) and flagellates for the higher trophic links of the aquatic foodwebs has been associated with the poor value of its lipid composition, especially that of polyunsaturated fatty acids (PUFA) and sterol [63,64,65]. 

PUFA are involved in the regulation of physiological processes by serving as precursors in the biosynthesis of bioactive molecules, and both PUFA and sterols are essential major membrane constituents of crustacean zooplankton, zoobenthos and fish [66,67]. According to Brett & Müller-Navarra [66], all herbivores convert the short-chain PUFA (*i.e.*, with <20 carbons) α-linolenic acid (18:3ω3) to long-chain PUFA (*i.e.*, with ≥20 carbons), such as EPA and DHA, albeit with different efficiency. In aquatic environments, most PUFA and sterols originate in the primary producers, essentially the photosynthetic autotrophs. Bacteria are generally poor in PUFA, especially long-chained PUFA. EPA-producing bacteria are not common in marine environments, yet Yazawa *et al.* [68] found 88 strains of bacteria capable of producing EPA out of 5000 strains screened. In fact, it was recently found that several marine bacteria contain EPA and DHA at levels as high as 25% of total membrane FA [69], and it is likely that the polyketide synthase (PKS) pathway for PUFA synthesis (that acts independently of FA elongase and desaturase activities to synthesize EPA directly) is widespread in marine bacteria [70]. Additionally, the culture conditions may determine the bacterial EPA content that is dependent on pH, temperature and other growth conditions [13]. Some crustacean species and nematodes may directly feed on bacteria and act as the first consumers of primary production, forming an important link between the basis of marine food webs and the higher trophic levels, like large metazoans and fish [71,72,73,74,75,76,77]. Direct feeding on bacteria may be crucial for crustacean species living close to hydrothermal vents, high-pressure low-temperature deep-sea habitats and permanently cold marine environments [78], or when bacteria is the sole item available [77]. It is worthy to note that in both deep-sea habitats and in the anoxic sediment of intertidal flats, bacteria may produce PUFA [13,79,80,81]. Nevertheless, the ecological role played by bacteria in aquatic environments cannot be dissociated from that of heterotrophic protists (HP). Heterotrophy is practiced by “mixotrophic” protists that include flagellated phytoplankton that may ingest bacteria or other protists [82] and by heterotrophic protists that do not possess permanent chloroplasts and rely on other organisms for nutrition [83]. HP are important consumers of bacteria and phytoplankton in oceanic food webs [83,84], act as regenerators of nutrients for further phytoplankton growth [85] and as a food resource for marine zooplankton [86,87]. Although HP have been described as early as the 1920s [88], their ecological importance was only fully appreciated when new analytical methods enabled their identification and quantification (e.g., epifluorescence microscopy and flow cytometry). The notion of a classical linear food chain in aquatic environments consisting of phytoplankton, zooplankton and fish predominated until the 1970s, and its replacement by the concept of a trophic web only gained ground in the 1980s [89,90,91]. In the trophic web concept, dissolved (DOM) and particulated organic matter (POM) consisting of detritus, heterotrophic bacteria and autotrophic phytoplankton are consumed by HP in a carbon-transfer pathway called the microbial food web. This microbial food web connects to the classical food chain that branches into a network of trophic links. Although picoplankton are responsible for the bulk of primary production in large parts of the marine environment (as well as lakes) [92,93,94], their small size makes them largely unavailable for direct consumption by crustacean zooplakton, which have difficulty in retaining these size particles in their filtering apparatus. In systems where microalgal species rich in high quality lipids (*i.e.*, PUFA) dominate the phytoplankton and can be directly grazed by crustacean zooplankton, the trophic transfer from autotrophs to crustaceans via HP can be considered both as a loss of carbon (*i.e.*, losses via respiration), as well as a loss of essential lipid compounds [95]. Nevertheless, HP feeding on nanophytoplankton and bacteria may biochemically enhance the quality of their prey, which led to the “trophic-upgrading” concept developed by Breteler *et al.* [96,97]. Thus, heterotrophic protists bridge the gap between the microbial loop and higher trophic levels by both repackaging their food and by increasing its nutritional value, which may be especially important when phytoplankton abundance is low or of reduced lipid quality and producers are dominated by prokaryotic picoplankton [97,98,99].

### 3.1. Transfer and Transformation of Bacterial Fatty Acids to Protists

The potentials for fatty acid and PUFA synthesis in HP are closely related to phylogenetic lineages (see Desvilettes & Bec and references therein [95]). Nevertheless, previous studies have identified two main factors affecting the PUFA composition of HP related to their habitat (marine *vs.* freshwater) and diet origin (bacteria *vs.* algae). While in freshwater HP ω6 FA dominate PUFA, marine HP contain high levels of ω3 highly unsaturated FA (HUFA), like EPA and DHA [100,101]. Both marine and freshwater HP feeding on algae have a higher content of ω3 PUFA than when feeding on bacteria [102,103], probably resulting from the higher availability of ω3 PUFA in algae. Most protists synthesize PUFA through a series of aerobic desaturations and elongations of the 16:0 and 18:0 acids produced by fatty acid synthase (FAS). Marine protists, namely thraustochytrids, are also able to produce PUFA using the PKS pathway and accumulate them in triacylglycerols [104,105]. Thraustochytrids, which are abundant in the marine foodweb, may be an important source of PUFA for the higher trophic levels [106]. In fact, thraustochytrids that may feed on bacteria are considered an alternative to fish oils as a source of long-chain PUFA [102] and are established candidates for commercial production of DHA [107].

### 3.2. “Transfer” of Sterol

Bacteria usually do not produce sterols, although there is evidence that some eubacteria are capable of synthesizing sterols *de novo* (e.g., *Methylococcus capsulatus* [108]). Crustaceans usually obtain their sterols directly via algae or through HP that have been feeding on algae. Some heterotrophic flagellates have the ability to synthesize sterols *de novo* [98], although ciliates seem to lack this ability. In the absence of dietary sterols, as when feeding on bacteria, ciliates produce the pentacyclic triterpenoid alcohol tetrahymanol or hopanoids, which serve as sterol surrogates in cell membranes (see Martin-Creuzburg and von Elert, and reference therein [109]). Some crustaceans (e.g., copepods) may incorporate tetrahymanol into their tissues, which enables them to maintain minimal egg production. Nevertheless, as Martin-Creuzburg and von Elert noted, it hasn’t been tested whether tetrahymanol or hopanoids improve the performance of crustaceans. Nevertheless, the production of sterols or functionally equivalent compounds, like tetrahymanol, by intermediary protozoans may improve carbon transfer efficiency via the microbial loop from nutritionally inadequate primary producers to metazoan grazers.

## 4. Conclusions

The ecological role played by bacteria in aquatic environments cannot be dissociated from that of heterotrophic protists. The efficiency of carbon transfer between bacteria and metazoans may be improved by intermediary protists, which elongate fatty acids and synthesize sterol or sterol surrogates.
